# 4-Chloro-*N*-(4-methyl­benzo­yl)benzene­sulfonamide

**DOI:** 10.1107/S1600536810019501

**Published:** 2010-05-29

**Authors:** P. A. Suchetan, B. Thimme Gowda, Sabine Foro, Hartmut Fuess

**Affiliations:** a Department of Chemistry, Mangalore University, Mangalagangotri 574 199, Mangalore, India; b Institute of Materials Science, Darmstadt University of Technology, Petersenstrasse 23, D-64287 Darmstadt, Germany

## Abstract

In the title compound, C_14_H_12_ClNO_3_S, the conformation of the N—H bond in the C—SO_2_—NH—C(O) segment is *anti* to the C=O bond. The mol­ecule is twisted at the S atom with a torsion angle of 69.0 (2)°. The dihedral angle between the sulfonyl benzene ring and the —SO_2_—NH—C—O segment is 77.2 (1)° and that between the sulfonyl and the benzoyl benzene rings is 89.5 (1)°. In the structure, mol­ecules are linked into chains *via* N—H⋯O hydrogen bonds, forming inversion dimers.

## Related literature

For background to our study of the effect of ring and side-chain substituents on the crystal structures of *N*-aromatic sulfonamides and for similar structures, see: Gowda *et al.* (2009 ▶); Suchetan *et al.* (2010**a* ▶,*b* ▶,c* ▶).
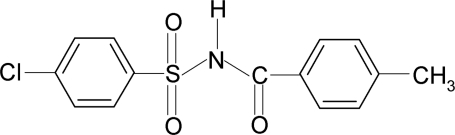


## Experimental

### 

#### Crystal data

C_14_H_12_ClNO_3_S
*M* *_r_* = 309.76
Orthorhombic, 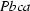

*a* = 13.719 (1) Å
*b* = 9.6781 (9) Å
*c* = 21.102 (2) Å
*V* = 2801.8 (4) Å^3^
*Z* = 8
Mo *K*α radiation
μ = 0.43 mm^−1^
*T* = 299 K
0.34 × 0.24 × 0.14 mm

#### Data collection

Oxford Diffraction Xcalibur diffractometer with a Sapphire CCD detector
Absorption correction: multi-scan (*CrysAlis RED*; Oxford Diffraction, 2009 ▶) *T*_min_ = 0.868, *T*_max_ = 0.943
6658 measured reflections
2840 independent reflections
1902 reflections with *I* > 2σ(*I*)
*R*_int_ = 0.018

#### Refinement

*R*[*F* ^2^ > 2σ(*F* ^2^)] = 0.046
*wR*(*F* ^2^) = 0.123
*S* = 1.02
2840 reflections
185 parameters
1 restraint
H atoms treated by a mixture of independent and constrained refinement
Δρ_max_ = 0.22 e Å^−3^
Δρ_min_ = −0.38 e Å^−3^

### 

Data collection: *CrysAlis CCD* (Oxford Diffraction, 2009 ▶); cell refinement: *CrysAlis RED* (Oxford Diffraction, 2009 ▶); data reduction: *CrysAlis RED*; program(s) used to solve structure: *SHELXS97* (Sheldrick, 2008 ▶); program(s) used to refine structure: *SHELXL97* (Sheldrick, 2008 ▶); molecular graphics: *PLATON* (Spek, 2009 ▶); software used to prepare material for publication: *SHELXL97*.

## Supplementary Material

Crystal structure: contains datablocks I, global. DOI: 10.1107/S1600536810019501/ds2033sup1.cif

Structure factors: contains datablocks I. DOI: 10.1107/S1600536810019501/ds2033Isup2.hkl

Additional supplementary materials:  crystallographic information; 3D view; checkCIF report

## Figures and Tables

**Table 1:** Hydrogen-bond geometry (Å, °)

*D*—H⋯*A*	*D*—H	H⋯*A*	*D*⋯*A*	*D*—H⋯*A*
N1—H1*N*⋯O3^i^	0.86 (1)	2.21 (1)	3.059 (3)	172 (2)

Symmetry code: (i) 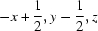.

**Fig. 1.**
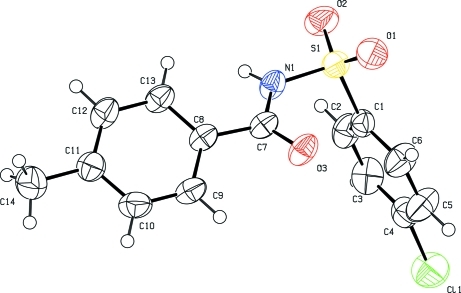
Molecular structure of the title compound, showing the atom- labelling scheme. Displacement ellipsoids are drawn at the 50% probability level.

**Fig. 2.**
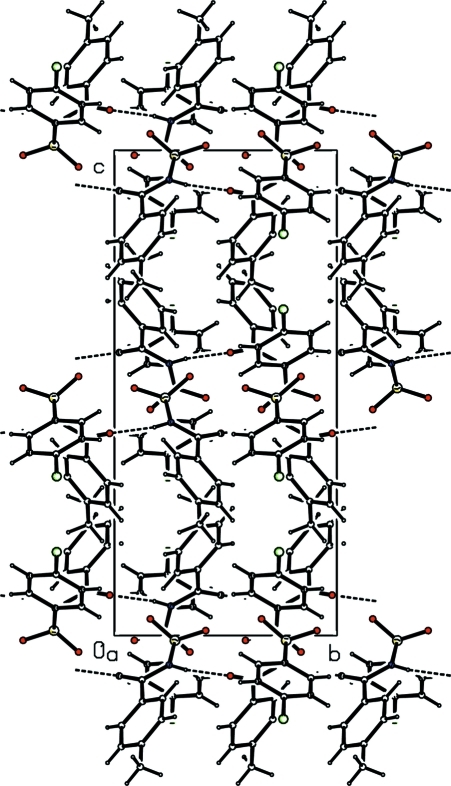
Molecular packing in the title compound. Hydrogen bonds are shown as dashed lines.

## supplementary crystallographic information

### Comment

As a part of studying the effect of ring and the side chain substituents on the crystal structures of *N*-aromatic sulfonamides(Gowda *et al.*, 2009; Suchetan *et al.*, 2010*a,b,c*), the structure of *N*-(4-methylbenzoyl)-4-chlorobenzenesulfonamide (I) has been determined. The conformation of the N—H bond in the C—SO_2_—NH—C(O) segment is *anti* to the C=O bond (Fig.1), similar to those observed in *N*-(benzoyl)benzenesulfonamide (II) (Gowda *et al.*, 2009), *N*-(benzoyl)-4-chlorobenzenesulfonamide (III) (Suchetan *et al.*, 2010*b*), *N*-(4-chlorobenzoyl)-4-methylbenzenesulfonamide (IV) (Suchetan *et al.*, 2010*a*), and *N*-(4-chlorobenzoyl)- 4-chlorobenzenesulfonamide (V)(Suchetan *et al.*, 2010*c*).

The molecules are twisted at the *S* atoms with the torsional angle of 69.0 (2)°, compared to the values of -66.9 (3)° in (II), -70.0 (2)° & 61.3 (2)° in the two molecules of (III), 67.1 (2)° (molecule 1) & 67.7 (2)° (molecule 2) in (IV) and 67.5 (3)° in (V).

The dihedral angle between the sulfonyl benzene ring and the —SO_2_—NH—C—O segment is 77.2 (1)°, compared to the values of 86.5 (1)° in (II), 72.0 (1)° and 77.3 (1)° in the two molecules of (III), 83.6 (1)° (molecule 1) & 81.0 (1)° (molecule 2) in (IV) and 79.0 (1)° in (V)

The dihedral angle between the sulfonyl and the benzoyl benzene rings is 89.5 (1)°, compared to the values of 80.3 (1) in (II), 62.8 (1)° (molecule 1) & 78.6 (1)° (molecule 2) in (III), 81.0 (1)° (molecule 1) and 76.3 (1)° (molecule 2) in (IV) and 85.6 (1)° in (V).

The packing of molecules linked by of N—H···O hydrogen bonds (Table 1) is shown in Fig. 2.

### Experimental

The title compound was prepared by refluxing a mixture of 4-methylbenzoic acid, 4-chlorobenzenesulfonamide and phosphorous oxy chloride for 3 h on a water bath. The resultant mixture was cooled and poured into ice cold water. The solid obtained was filtered, washed thoroughly with water and then dissolved in sodium bicarbonate solution. The compound was later reprecipitated by acidifying the filtered solution with dilute HCl. It was filtered, dried and recrystallized.

Rod like colourless single crystals of the title compound used in X-ray diffraction studies were obtained by slow evaporation of its toluene solution at room temperature.

### Refinement

The H atom of the NH group was located in a difference map and later restrained to N—H = 0.86 (1) %A. The other H atoms were positioned with idealized geometry using a riding model with C—H = 0.93–0.96 Å. All H atoms were refined with isotropic displacement parameters (set to 1.2 times of the *U*_eq_ of the parent atom).

### Crystal data

**Table:**

C_14_H_12_ClNO_3_S	*F*(000) = 1280
*M**_r_* = 309.76	*D*_x_ = 1.469 Mg m^−^^3^
Orthorhombic, *P**b**c**a*	Mo *K*α radiation, λ = 0.71073 Å
Hall symbol: -P 2ac 2ab	Cell parameters from 2077 reflections
*a* = 13.719 (1) Å	θ = 2.6–27.8°
*b* = 9.6781 (9) Å	µ = 0.43 mm^−^^1^
*c* = 21.102 (2) Å	*T* = 299 K
*V* = 2801.8 (4) Å^3^	Rod, colourless
*Z* = 8	0.34 × 0.24 × 0.14 mm

### Data collection

**Table:**

Oxford Diffraction Xcalibur diffractometer with a Sapphire CCD detector	2840 independent reflections
Radiation source: fine-focus sealed tube	1902 reflections with *I* > 2σ(*I*)
graphite	*R*_int_ = 0.018
Rotation method data acquisition using ω and phi scans	θ_max_ = 26.4°, θ_min_ = 2.8°
Absorption correction: multi-scan (*CrysAlis RED*; Oxford Diffraction, 2009)	*h* = −13→17
*T*_min_ = 0.868, *T*_max_ = 0.943	*k* = −7→12
6658 measured reflections	*l* = −26→15

### Refinement

**Table:**

Refinement on *F*^2^	Primary atom site location: structure-invariant direct methods
Least-squares matrix: full	Secondary atom site location: difference Fourier map
*R*[*F*^2^ > 2σ(*F*^2^)] = 0.046	Hydrogen site location: inferred from neighbouring sites
*wR*(*F*^2^) = 0.123	H atoms treated by a mixture of independent and constrained refinement
*S* = 1.02	*w* = 1/[σ^2^(*F*_o_^2^) + (0.0556*P*)^2^ + 1.3127*P*] where *P* = (*F*_o_^2^ + 2*F*_c_^2^)/3
2840 reflections	(Δ/σ)_max_ = 0.001
185 parameters	Δρ_max_ = 0.22 e Å^−^^3^
1 restraint	Δρ_min_ = −0.38 e Å^−^^3^

### Special details

**Table:**

Experimental. CrysAlis RED (Oxford Diffraction, 2009) Empirical absorption correction using spherical harmonics, implemented in SCALE3 ABSPACK scaling algorithm.
Geometry. All e.s.d.'s (except the e.s.d. in the dihedral angle between two l.s. planes) are estimated using the full covariance matrix. The cell e.s.d.'s are taken into account individually in the estimation of e.s.d.'s in distances, angles and torsion angles; correlations between e.s.d.'s in cell parameters are only used when they are defined by crystal symmetry. An approximate (isotropic) treatment of cell e.s.d.'s is used for estimating e.s.d.'s involving l.s. planes.
Refinement. Refinement of *F*^2^ against ALL reflections. The weighted *R*-factor *wR* and goodness of fit *S* are based on *F*^2^, conventional *R*-factors *R* are based on *F*, with *F* set to zero for negative *F*^2^. The threshold expression of *F*^2^ > σ(*F*^2^) is used only for calculating *R*-factors(gt) *etc*. and is not relevant to the choice of reflections for refinement. *R*-factors based on *F*^2^ are statistically about twice as large as those based on *F*, and *R*- factors based on ALL data will be even larger.

### Fractional atomic coordinates and isotropic or equivalent isotropic displacement parameters (Å^2^)

**Table:**

	*x*	*y*	*z*	*U*_iso_*/*U*_eq_	
C1	0.01547 (17)	0.2340 (2)	0.44377 (11)	0.0399 (6)	
C2	−0.0141 (2)	0.1166 (3)	0.41247 (14)	0.0546 (7)	
H2	0.0228	0.0362	0.4151	0.066*	
C3	−0.0983 (2)	0.1187 (3)	0.37717 (15)	0.0594 (8)	
H3	−0.1187	0.0399	0.3557	0.071*	
C4	−0.15186 (19)	0.2375 (3)	0.37395 (13)	0.0498 (7)	
C5	−0.1248 (2)	0.3538 (3)	0.40560 (15)	0.0647 (9)	
H5	−0.1630	0.4331	0.4037	0.078*	
C6	−0.0399 (2)	0.3523 (3)	0.44044 (14)	0.0601 (8)	
H6	−0.0200	0.4315	0.4618	0.072*	
C7	0.23281 (17)	0.3764 (2)	0.40867 (12)	0.0396 (6)	
C8	0.31666 (17)	0.3739 (2)	0.36415 (12)	0.0389 (6)	
C9	0.31635 (19)	0.4681 (3)	0.31449 (13)	0.0477 (7)	
H9	0.2653	0.5307	0.3105	0.057*	
C10	0.3915 (2)	0.4689 (3)	0.27110 (13)	0.0543 (7)	
H10	0.3895	0.5311	0.2375	0.065*	
C11	0.47023 (19)	0.3788 (3)	0.27635 (13)	0.0485 (7)	
C12	0.47077 (19)	0.2883 (3)	0.32716 (14)	0.0505 (7)	
H12	0.5236	0.2291	0.3325	0.061*	
C13	0.39507 (17)	0.2836 (2)	0.37016 (13)	0.0452 (6)	
H13	0.3965	0.2200	0.4032	0.054*	
C14	0.5521 (2)	0.3835 (4)	0.22922 (14)	0.0688 (9)	
H14A	0.6100	0.4170	0.2496	0.083*	
H14B	0.5349	0.4441	0.1950	0.083*	
H14C	0.5636	0.2923	0.2129	0.083*	
N1	0.21363 (15)	0.25385 (19)	0.43927 (10)	0.0426 (5)	
H1N	0.2442 (17)	0.1798 (17)	0.4294 (12)	0.051*	
O1	0.11997 (14)	0.3386 (2)	0.53406 (9)	0.0630 (6)	
O2	0.13812 (14)	0.0896 (2)	0.51002 (10)	0.0652 (6)	
O3	0.18237 (12)	0.47861 (16)	0.41670 (9)	0.0508 (5)	
Cl1	−0.25653 (6)	0.24046 (10)	0.32788 (4)	0.0778 (3)	
S1	0.12257 (5)	0.22796 (7)	0.48971 (3)	0.0467 (2)	

### Atomic displacement parameters (Å^2^)

**Table:**

	*U*^11^	*U*^22^	*U*^33^	*U*^12^	*U*^13^	*U*^23^
C1	0.0343 (12)	0.0428 (13)	0.0427 (13)	−0.0006 (11)	0.0059 (11)	0.0013 (11)
C2	0.0459 (16)	0.0424 (15)	0.076 (2)	0.0001 (12)	0.0084 (15)	−0.0008 (14)
C3	0.0526 (17)	0.0527 (17)	0.073 (2)	−0.0120 (14)	0.0018 (16)	−0.0128 (15)
C4	0.0354 (13)	0.0656 (19)	0.0484 (15)	−0.0080 (13)	0.0021 (12)	−0.0025 (14)
C5	0.0543 (18)	0.0612 (19)	0.079 (2)	0.0170 (15)	−0.0166 (17)	−0.0161 (16)
C6	0.0596 (19)	0.0487 (16)	0.072 (2)	0.0117 (14)	−0.0163 (16)	−0.0231 (15)
C7	0.0333 (12)	0.0321 (12)	0.0533 (15)	−0.0024 (10)	−0.0082 (11)	−0.0003 (11)
C8	0.0307 (12)	0.0315 (12)	0.0546 (15)	−0.0052 (10)	−0.0063 (12)	0.0027 (11)
C9	0.0404 (14)	0.0418 (14)	0.0610 (17)	0.0024 (11)	−0.0080 (14)	0.0111 (13)
C10	0.0506 (16)	0.0581 (17)	0.0543 (17)	−0.0041 (14)	−0.0065 (14)	0.0172 (14)
C11	0.0400 (14)	0.0534 (16)	0.0521 (16)	−0.0047 (12)	−0.0018 (13)	0.0007 (13)
C12	0.0374 (13)	0.0434 (14)	0.0708 (18)	0.0016 (11)	0.0027 (13)	0.0051 (14)
C13	0.0381 (14)	0.0341 (13)	0.0634 (17)	−0.0007 (11)	−0.0008 (12)	0.0102 (12)
C14	0.0534 (17)	0.092 (2)	0.061 (2)	−0.0003 (17)	0.0042 (16)	0.0058 (17)
N1	0.0375 (11)	0.0293 (10)	0.0609 (14)	0.0022 (9)	0.0068 (10)	0.0059 (10)
O1	0.0613 (13)	0.0790 (14)	0.0488 (11)	0.0100 (11)	−0.0077 (10)	−0.0115 (10)
O2	0.0522 (12)	0.0624 (12)	0.0811 (14)	0.0026 (9)	0.0055 (11)	0.0353 (11)
O3	0.0428 (10)	0.0329 (9)	0.0768 (13)	0.0056 (8)	0.0008 (10)	0.0044 (9)
Cl1	0.0498 (4)	0.1078 (7)	0.0758 (6)	−0.0148 (4)	−0.0150 (4)	0.0022 (5)
S1	0.0408 (4)	0.0505 (4)	0.0489 (4)	0.0031 (3)	0.0019 (3)	0.0095 (3)

### Geometric parameters (Å, °)

**Table:**

C1—C2	1.375 (3)	C9—C10	1.379 (4)
C1—C6	1.376 (3)	C9—H9	0.9300
C1—S1	1.761 (3)	C10—C11	1.393 (4)
C2—C3	1.375 (4)	C10—H10	0.9300
C2—H2	0.9300	C11—C12	1.384 (4)
C3—C4	1.366 (4)	C11—C14	1.500 (4)
C3—H3	0.9300	C12—C13	1.380 (4)
C4—C5	1.360 (4)	C12—H12	0.9300
C4—Cl1	1.734 (3)	C13—H13	0.9300
C5—C6	1.377 (4)	C14—H14A	0.9600
C5—H5	0.9300	C14—H14B	0.9600
C6—H6	0.9300	C14—H14C	0.9600
C7—O3	1.219 (3)	N1—S1	1.660 (2)
C7—N1	1.376 (3)	N1—H1N	0.856 (10)
C7—C8	1.485 (3)	O1—S1	1.423 (2)
C8—C9	1.389 (3)	O2—S1	1.4224 (19)
C8—C13	1.392 (3)		
			
C2—C1—C6	120.0 (2)	C9—C10—C11	121.6 (3)
C2—C1—S1	118.9 (2)	C9—C10—H10	119.2
C6—C1—S1	121.1 (2)	C11—C10—H10	119.2
C1—C2—C3	119.7 (3)	C12—C11—C10	117.5 (3)
C1—C2—H2	120.1	C12—C11—C14	121.9 (3)
C3—C2—H2	120.1	C10—C11—C14	120.6 (3)
C4—C3—C2	119.4 (3)	C13—C12—C11	121.8 (2)
C4—C3—H3	120.3	C13—C12—H12	119.1
C2—C3—H3	120.3	C11—C12—H12	119.1
C5—C4—C3	121.7 (3)	C12—C13—C8	120.1 (2)
C5—C4—Cl1	119.2 (2)	C12—C13—H13	120.0
C3—C4—Cl1	119.1 (2)	C8—C13—H13	120.0
C4—C5—C6	119.0 (3)	C11—C14—H14A	109.5
C4—C5—H5	120.5	C11—C14—H14B	109.5
C6—C5—H5	120.5	H14A—C14—H14B	109.5
C1—C6—C5	120.2 (3)	C11—C14—H14C	109.5
C1—C6—H6	119.9	H14A—C14—H14C	109.5
C5—C6—H6	119.9	H14B—C14—H14C	109.5
O3—C7—N1	121.7 (2)	C7—N1—S1	125.09 (17)
O3—C7—C8	122.7 (2)	C7—N1—H1N	121.0 (18)
N1—C7—C8	115.5 (2)	S1—N1—H1N	113.4 (18)
C9—C8—C13	118.9 (2)	O2—S1—O1	120.95 (13)
C9—C8—C7	117.7 (2)	O2—S1—N1	102.86 (11)
C13—C8—C7	123.5 (2)	O1—S1—N1	109.09 (12)
C10—C9—C8	120.2 (2)	O2—S1—C1	108.79 (12)
C10—C9—H9	119.9	O1—S1—C1	108.44 (12)
C8—C9—H9	119.9	N1—S1—C1	105.66 (11)
			
C6—C1—C2—C3	0.9 (4)	C9—C10—C11—C14	−178.8 (3)
S1—C1—C2—C3	179.0 (2)	C10—C11—C12—C13	2.0 (4)
C1—C2—C3—C4	−0.3 (4)	C14—C11—C12—C13	−179.5 (3)
C2—C3—C4—C5	−1.0 (5)	C11—C12—C13—C8	−1.8 (4)
C2—C3—C4—Cl1	178.5 (2)	C9—C8—C13—C12	−0.1 (4)
C3—C4—C5—C6	1.6 (5)	C7—C8—C13—C12	−179.4 (2)
Cl1—C4—C5—C6	−177.9 (2)	O3—C7—N1—S1	0.0 (4)
C2—C1—C6—C5	−0.2 (4)	C8—C7—N1—S1	−178.24 (17)
S1—C1—C6—C5	−178.3 (2)	C7—N1—S1—O2	−177.0 (2)
C4—C5—C6—C1	−1.0 (5)	C7—N1—S1—O1	−47.4 (2)
O3—C7—C8—C9	−24.1 (4)	C7—N1—S1—C1	69.0 (2)
N1—C7—C8—C9	154.1 (2)	C2—C1—S1—O2	−26.1 (2)
O3—C7—C8—C13	155.3 (3)	C6—C1—S1—O2	152.0 (2)
N1—C7—C8—C13	−26.5 (3)	C2—C1—S1—O1	−159.4 (2)
C13—C8—C9—C10	1.7 (4)	C6—C1—S1—O1	18.6 (3)
C7—C8—C9—C10	−178.9 (2)	C2—C1—S1—N1	83.7 (2)
C8—C9—C10—C11	−1.5 (4)	C6—C1—S1—N1	−98.2 (2)
C9—C10—C11—C12	−0.4 (4)		

### Hydrogen-bond geometry (Å, °)

**Table:**

*D*—H···*A*	*D*—H	H···*A*	*D*···*A*	*D*—H···*A*
N1—H1N···O3^i^	0.86 (1)	2.21 (1)	3.059 (3)	172 (2)

Symmetry codes: (i) −*x*+1/2, *y*−1/2, *z*.
